# Gene expression patterns and environmental enrichment-induced effects in the hippocampi of mice suggest importance of Lsamp in plasticity

**DOI:** 10.3389/fnins.2015.00205

**Published:** 2015-06-08

**Authors:** Indrek Heinla, Este Leidmaa, Karina Kongi, Airi Pennert, Jürgen Innos, Kaarel Nurk, Triin Tekko, Katyayani Singh, Taavi Vanaveski, Riin Reimets, Merle Mandel, Aavo Lang, Kersti Lilleväli, Allen Kaasik, Eero Vasar, Mari-Anne Philips

**Affiliations:** ^1^Department of Physiology, Institute of Biomedicine and Translational Medicine, University of TartuTartu, Estonia; ^2^Stress Neurobiology and Neurogenetics, Max Planck Institute of PsychiatryMunich, Germany; ^3^Department of Pharmacology, Institute of Biomedicine and Translational Medicine, University of TartuTartu, Estonia

**Keywords:** *Lsamp*, enrichment, isolation, hippocampus, mice, *Bdnf*, B6, 129Sv

## Abstract

*Limbic system associated membrane protein* (*Lsamp*) gene is involved in behavioral adaptation in social and anxiogenic environments and has been associated with a broad spectrum of psychiatric diseases. Here we studied the activity of alternative promoters of *Lsamp* gene in mice in three rearing conditions (standard housing, environmental enrichment and social isolation) and in two different genetic backgrounds (129S6/SvEv and C57BL/6). Isolation had no effect on the expression levels of *Lsamp*. Environmental enrichment elevated the expression levels of *Lsamp* 1b transcript specifically in the hippocampus in B6 mice, and the same tendency existed across both mouse lines and both transcripts. Furthermore, we showed that the density of cells exhibiting 1b promoter activity is remarkably higher in the subgranular zone of the dentate gyrus in the hippocampal formation which is a specific area of enrichment-induced neurogenesis in adult rodents. On the contrary to 1b, 1a promoter is selectively active in the pyramidal and granule cell layers. We provide evidence that *Lsamp* modulates enrichment-induced activation of *Bdnf* as the enrichment-induced elevation of *Bdnf* in the hippocampus is significantly diminished in *Lsamp*-deficient mice; furthermore, a significant correlation was found between the expression levels of *Lsamp* and *Bdnf* transcripts in the hippocampus and frontal cortex. Significant strain differences in *Lsamp* expression were detected in the hippocampus, frontal cortex and thalamus that could be related to the different behavioral phenotype of B6 and 129Sv mice. Our data provides further evidence that LSAMP is implicated in the hippocampal connectivity and plasticity thereby modulating adaptability in changing environments.

## Introduction

A growing field of social genomics research has begun to identify the specific types of genes that are subject to social-environmental regulation, the neural, and molecular mechanisms that mediate the effects social processes have on gene expression (Slavich and Cole, 2013). Enrichment and social isolation are the most common environmental manipulations used in laboratory rodent housing. In certain study designs rodents must stay in isolation; e.g., studies which require surgical intervention (Bailey and Crawley, 2009) or investigation of feeding behavior (where food intake per animal has to be measured with great precision) (Ellacott et al., 2010). At the same time different kinds of enrichment equipment is applied to standard rodent laboratory housing conditions worldwide (Heinla et al., 2014). Therefore, it is vital to understand the molecular and functional impact of these environmental manipulations on the brain and behavior. Environmental enrichment is known to both profoundly affect the central nervous system at the transcriptome level (Rampon et al., 2000) and influence the fine structural anatomy of neural networks (Kempermann et al., 1997; Freund et al., 2013) during the critical developmental period and during adulthood (Baroncelli et al., 2010). Social isolation and rejection can influence the activity of a broad set of genes (Bibancos et al., 2007; Sestito et al., 2011) and cause permanent changes in the brain and behavior throughout lifespan (Fone and Porkess, 2008). The discovery that social-environmental factors can substantially alter the expression of meaningfully identified gene profiles represents a paradigm shift in thinking about gene-environment interactions (Slavich and Cole, 2013).

Accumulating evidence suggests that *Limbic system associated membrane protein* (*Lsamp*) gene expression is sensitive to changes in external social and environmental conditions and it could mediate neural plasticity. LSAMP is a neural cell adhesion molecule expressed on the neuronal dendrites and somata (Zacco et al., 1990). The anatomical distribution of LSAMP is controlled by complex regulation of alternative 1a and 1b promoters. *Lsamp* 1a promoter is more expressed in “classical” limbic system areas such as hippocampus, amygdala, insular cortex etc.; 1b is expressed in the areas that process sensory information (Philips et al., 2015). The impact of LSAMP protein on neurite outgrowth (Mann et al., 1998; Gil et al., 2002) and neuronal connectivity has been established in a wide spectrum of psychiatric disorders in humans (Behan et al., 2009; Koido et al., 2012). In mice, lack of LSAMP protein leads to inability to adapt or react to novel environments or stressful environmental manipulations in an evolutionarily sustainable way (Catania et al., 2008; Innos et al., 2011, 2013). *Lsamp*-deficient mice are less sensitive to social isolation which is usually stressful for wild-type mice; furthermore, inadequately reduced anxiety reaction in potentially threatening situations is amplified if *Lsamp*-deficient mice have been reared in an enriched environment (Innos et al., 2012).

LSAMP protein has been shown to increase synaptogenesis in the hippocampal neurons *in vitro* (Hashimoto et al., 2009) indicating its role in plasticity. Furthermore, loss of LSAMP *in vivo* results in altered synaptic transmission and impaired plasticity in adult hippocampus (Qiu et al., 2010). Synaptic plasticity has been considered to be one of the main mechanisms responsible for the neuronal changes that occur in response to complex stimulation by enriched environment (van Praag et al., 2000). *Lsamp* gene expression, however, has never been studied in different environments. To include genetic background which is known to influence the phenotypes caused by single genes (Navarro et al., 2012) we used two inbred mouse lines. B6 and 129Sv are common inbred strains used in behavioral neuroscience which are of special interest to transgenic research (Heinla et al., 2014). To shed light on gene expression regulation influenced by complex interaction between environment and individuals' genetic background, our study included six experimental groups: two different inbred mouse lines living in enriched, standard, or individual housing. Additionally, the alternative activity of *Lsamp* 1a and 1b promoters was analyzed in six different brain areas.

## Materials and methods

### Animals

C57BL/6 Bkl—ScanburAB, Sollentuna, Sweden; 129S6/SvEv/Tac—Taconic Europe, Bomholt, Denmark; *Lsamp*-deficient mice—produced in the Institute of Biomedicine and Translational Medicine, University of Tartu, Estonia.

Detailed description of the creation of *Lsamp*-deficient mice with LacZ transgene can be found in Innos et al. (2011). Briefly, exon 1b of mouse *Lsamp* gene was replaced by an in-frame NLSLacZNeo cassette resulting in insertion of gene encoding beta-galactosidase immediately after *Lsamp* 1b promoter. As a result, these mice could not express functional LSAMP protein from either of the promoters.

Breeding and housing were conducted in the Institute of Biomedicine and Translational Medicine, University of Tartu. Male mice were housed under a 12-h light/dark cycle with lights on at 7:00 a.m. All strains were housed in their respective home cages. The animals had free access to food and water except during testing. The bedding (aspen chips) and nesting material (aspen wool) were changed once a week. Mice were held in groups of 7–8 animals per cage, except for the individually housed mice.

### Environmental enrichment (EE) and individual housing (IH)

After weaning at 3 weeks, mice were randomly allocated to either standard, enriched, or individual housing conditions for 7 weeks (129Sv and B6) or 8 weeks (*Lsamp*-deficient mice and their wild-type littermates) before the start of the experiments.

Standard housing consisted of standard laboratory cages (425 × 266 × 155 mm) with bedding and nesting material.

Mice in the environmental enrichment group were housed in larger cages (595 × 380 × 200 mm) containing double amount of nesting material, stainless steel running wheels, aspen houses, igloos, tubes, or labyrinths, which were changed and repositioned once a week. Each enriched cage always had five items, always including at least one running wheel and either a house or an igloo for shelter.

Individually housed mice lived in smaller (220 × 160 ×140 mm) cages with standard bedding and a small amount of nesting material.

### Hippocampal stainings

The 1a and 1b promoter specific stainings have been performed as described previously (Philips et al., 2015). Briefly, the distribution of *Lsamp* 1a transcript and summarized expression of the *Lsamp* transcripts was investigated by non-radioactive *in situ* RNA hybridization analysis; *Lsamp*-deficient mice with a LacZ transgene (Innos et al., 2011) was used for visualizing the anatomical distribution of *Lsamp* 1b promoter activity. New stainings and higher resolution images of the hippocampal area were performed to enable precise analysis of the anatomical distribution of the activity of *Lsamp* 1a and 1b promoters in the sub regions of hippocampal sections in mouse. A comprehensive analysis of the reliability of using alternative staining methods for *Lsamp* 1a and 1b promoter activities has been published previously in Philips et al. (2015).

In BrdU and X-Gal co-stainings, male adult *Lsamp* deficient mouse received two injections of 5-bromodeoxyuridine (BrdU; 100 μg/g) with 2-h interval and was sacrificed 24 h after the last injection. X-Gal staining was performed as in Philips et al. (2015). BrdU incorporation was detected immunohistochemically using monoclonal rat anti-BrdU (AbDSerotec), biotinylated donkey anti-rat (Dako) antibodies, and Vectastain Elite ABC Kit (Vector Laboratories). Peroxydase reaction was detected by DAB detection kit (Vector Laboratories).

Dissociated primary hippocampal neuronal cultures were prepared according to Chatterjee and Sikdar (2014) from the whole hippocampus of 0–2 days old mouse pups (*Lsamp* knockout). Hippocampus was digested in papain/DNase solution and neuronal cells were suspended in culture media consisting Dulbecco's modified Eagle's medium F12 HAM supplemented with N1, 10% fetal bovine serum and 1% antibiotic antimycotic. Cells were plated on 0.1 mg/mL poly-D lysine coated white microwell plates (96 F Nuclon Delta, Nunc) at a density of 20,000–50,000 cells in 2 ml media. For X-Gal staining primary hippocampal neurons were fixed with 2% PFA in PB Buffer for 15 min and washed three times in PBS for 15 min. Neurons were stained overnight with X-Gal solution. After staining again neurons were treated with 4% PFA for complete fixation.

### qRT-PCR analysis in mouse brain areas

The C57BL/6 (*n* = 3 × 8) and 129S6/SvEv (*n* = 3 × 8) mice were sacrificed by decapitation 10 days after the last experiment at the age of 15 weeks and brain regions of interest were collected into Eppendorf tubes and kept at 80°C. *Lsamp* mRNA level was determined by quantitative real-time PCR (qRT-PCR) in six brain regions. Total RNA was extracted individually from each brain structure by using Trizol® Reagent (Invitrogen, USA) according to the manufacturer's protocol. First strand cDNA was synthesized by using Random hexamerprimermix (Applied Biosystems) and SuperScript™ III Reverse Transcriptase (Invitrogen, USA). TaqMan Assay was designed for the detection of 1a and 1b specific transcripts. FAM-MGB-probe AACCGAGGCACGGACAAC was used with universal reverse primer combined with alternative forward oligos specific for either 1a allele or 1b allele. TaqMan® Universal PCR Master Mix was used in the ABI Prism 7900HT Sequence Detection System (Applied Biosystems, USA). Reactions were carried out in 10 μl reaction volumes in four replicates. *Bdnf* and *synaptophysin* mRNA levels were determined by quantitative real-time PCR (qRT-PCR) by using the predesigned Taqman Gene Expression Assays (Applied Biosystems): Mm01334042 m1(*Bdnf*) and Mm00436850_m1 (*synaptophysin*; the assay previously used in Abel and Rissman (2013). The expressional results for *Bdnf* that have been described in Heinla et al. (2014) were used as a control of study design.

### Data analysis and statistics

Mean values and S.E.M. are presented in the figures. All data were analyzed using Statistical version 10 (StatSoft, Inc., USA). Factorial ANOVA (strain × environment as grouping variables) was performed to compare the mRNA expression of experimental groups. For comparing 1a and 1b promoter expressions, repeated measures ANOVA (strain and environment as grouping variables and promoters as within subject factor) was used. Tukey HSD *post-hoc* analysis was used when applicable after statistically significant ANOVA.

The analysis of qRT-PCR data was performed as described previously by Raud et al. (2009). Briefly, qRT-PCR data in figures is presented on a linear scale, calculated as *2*^−Δ*CT*^, where Δ*CT* is the difference in cycle threshold (CT) between the target gene (*Lsamp*) and housekeeper gene *Hprt-1* (VIC-MGB). Reported correlations were calculated using Pearson's Product-Moment correlation method.

## Results

### The effect of environment and genetic background on *Lsamp* gene expression

**Figure 1:**
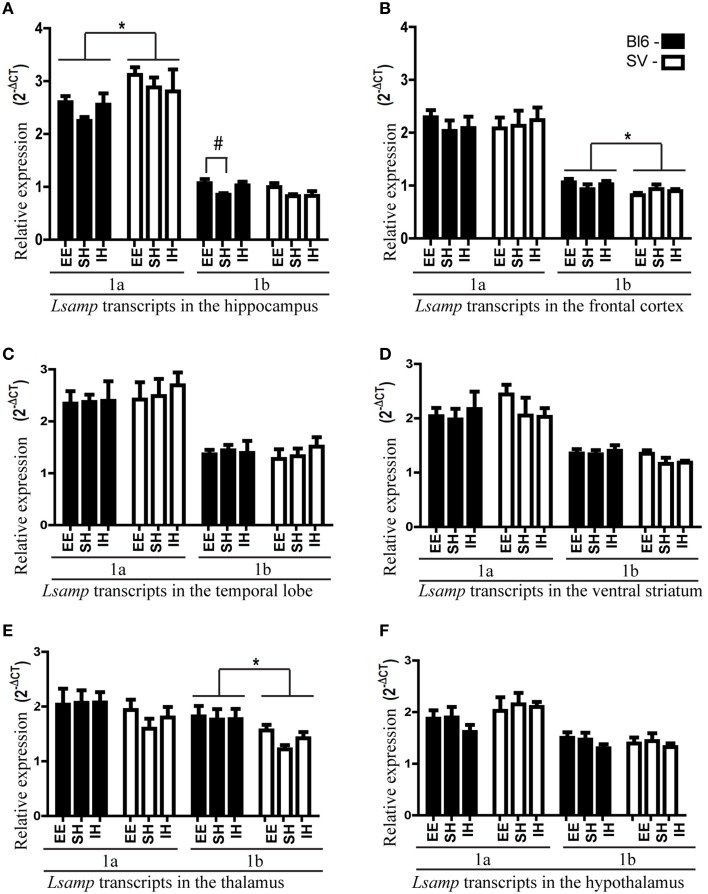
***Lsamp*** **1a and 1b promoter mRNA expression in 6 different brain structures of mice raised in three different environmental conditions**. The data has been presented separately for the mice with B6 and 129 background. **(A)** In the hippocampus environmental enrichment elevates *Lsamp* expression; # *p* < 0.05 (Tukey HSD test after significant ANOVA). 1a expression is higher in 129Sv mice compared to B6; ^*^p < 0.05 (Main effect of genotype in ANOVA). **(B)** In the frontal cortex 1b promoter is more prominent in B6 mice compared to 129Sv; ^*^p < 0.05 (Main effect of genotype in ANOVA). **(C)** In the temporal lobe there were no significant differences in *Lsamp* promoter expression. **(D)** In the ventral striatum there were no significant differences in *Lsamp* promoter expression. **(E)** In the thalamus 1b promoter is more prominent in B6 mice compared to 129Sv; ^*^p < 0.01 (Main effect of genotype in ANOVA). **(F)** In the hypothalamus there were no significant differences in *Lsamp* promoter expression.

In general, the expression levels of *Lsamp* transcripts in different brain areas were stable regardless of different rearing conditions. A remarkable environmental effect was the increase of *Lsamp* transcripts in mice raised in enriched environment. *Lsamp* 1b transcript level was significantly elevated in the hippocampal area of B6 mice [Figure 1A, B6 standard housing vs. enriched environment *F*_(2, 20)_ = 4.47; *p* < 0.05]. The trend of enrichment-induced elevation of both 1a and 1b transcripts exists in 129Sv and B6 background but is only statistically significant in the case of 1b promoter in B6 mice [Figure 1A, *F*_(2, 42)_ = 3.98; *p* < 0.05]; in pooled backgrounds the effect is again significant in the case of 1b [*Lsamp* 1a *F*_(2, 45)_ = 0.83, *p* = 0.44; *Lsamp* 1b *F*_(2, 45)_ = 3.82 *p* < 0.05]. There were no other enrichment- or isolation-induced effects on *Lsamp* expression.

We detected strain differences between B6 and 129Sv mice in *Lsamp* expression levels. *Lsamp* 1a transcript was higher in the hippocampal area of 129Sv mice [Figure 1A; *F*_(1, 46)_ = 6.92, *p* < 0.05] while *Lsamp* 1b had higher expression levels in the frontal cortex [Figure 1B; *F*_(1, 46)_ = 4.92 *p* < 0.05] and thalamus [Figure 1E; *F*_(1, 46)_ = 10.05 *p* < 0.01] of B6 mice.

### The anatomical distribution of *Lsamp* 1a and 1b promoter activity

**Figure 2:**
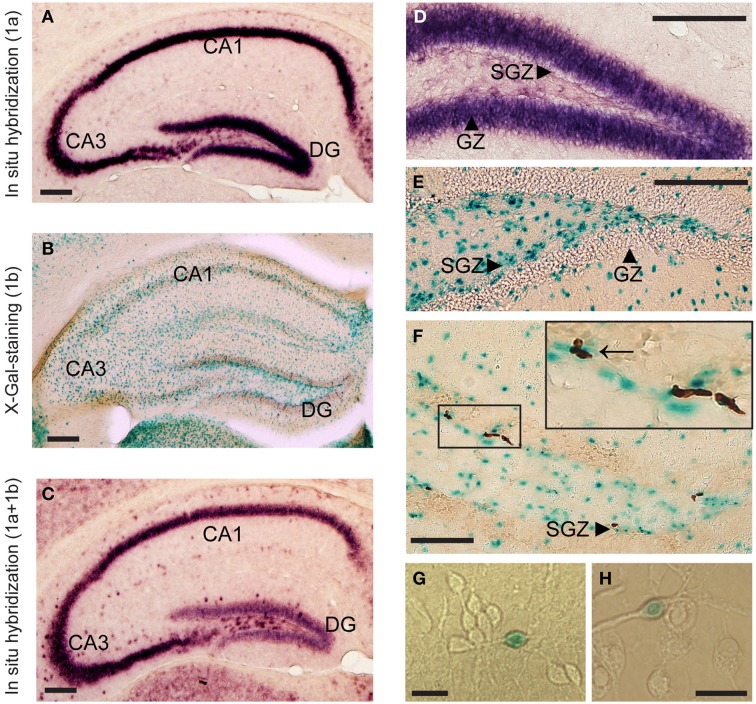
**The anatomical distribution of alternative promoter activities of** ***Lsamp*** **gene**. mRNA *in situ* hybridization indicates *Lsamp* 1a **(A,D)** and summarized (1a plus 1b); **(C)** promoter activity, X-Gal staining indicates 1b activity **(B,E,F)** and BrdU incorporation indicates proliferation **(F)**. Arrow on **(F)** points to X-Gal positive cells that show also BrdU staining. Arrowheads on **(D–F)** point to the specific compartments of hippocampal formation. **(G,H)**: X-Gal positive cells in 10-day old **(G)** and in 21-day old **(H)** hippocampal culture. Abbreviations: DG, dentate gyrus; GZ, granular zone; SGZ, subgranular zone. Scale bars represent 0.2 mm **(A–F)** and 30 μm **(G,H)**.

1a promoter had significantly higher expression levels in all six brain areas compared to 1b transcript [Figures 1A–F; difference is significant in all tissues: *p* < 0.001]. The relative expression levels of *Lsamp* 1a and 1b transcripts in six different brain areas were well in line with the data from our previous analysis about the anatomical distribution of alternative promoters of *Lsamp* gene (Philips et al., 2015). The expression level of *Lsamp* 1a promoter was highest in the hippocampus compared to other brain areas (Figure 1). In the mouse hippocampal formation 1a promoter was almost exclusively expressed in the pyramidal cell layer in CA1, CA2, and CA3 regions (Figure 2A) and in the granule cell layer (GL) of the dentate gyrus (DG, Figure 2D). There were a few 1a positive cells spread all over hilus. In the mouse hippocampal formation 1b promoter was sparsely expressed all over the structure (Figure 2B). In the DG, there were notably more concentrated 1b signals in the subgranular zone (SGZ) and 1b staining was nearly missing in the granule cell layer (Figure 2E). Summarized staining reveals strong expression of *Lsamp* in the pyramidal cell layer in CA1, CA2, and CA3 regions and evidently less intensive staining in the GL of the DG (Figure 2C), confirming that both promoters of the *Lsamp* gene are active in the pyramidal cells of the hippocampus but only 1a promoter is active in the granule cell layer in the gyrus dentatus in both B6 and 129Sv mice. The analysis of neurogenesis in the adult mouse DG showed a remarkable spatial overlap between the expressional activity of *Lsamp* 1b (X-Gal staining) transcript in BrdU positive proliferating cells. Namely, both stainings were prominent in the subgranular zone of DG, moreover many X-Gal positive cells also showed clearly BrdU staining (arrow in Figure 2F). Still, not all proliferating cells showed 1b promoter activity. In the primary culture analysis, all the cells that displayed X-Gal staining were morphologically clearly identified as neurons in both 10-day (Figure 2G) and 21-day (Figure 2H) hippocampal cultures and approximately 8–10% of all neurons were X-Gal positive.

### The effect of environment on *Bdnf* and *Synaptophysin* gene expression

**Figure 3:**
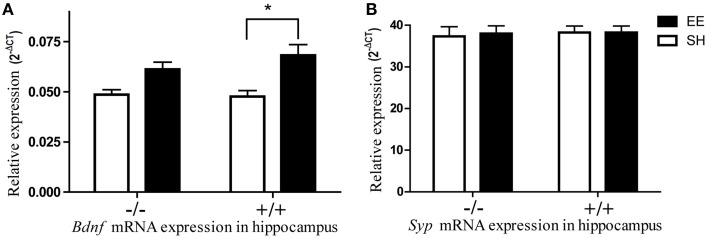
**The influence of environmental enrichment on the** ***Bdnf*** **and Syp mRNA expression in the hippocampus of** ***Lsamp*** **deficient mice and their wild-type littermates**. **(A)** Environmental enrichment increases *Bdnf* mRNA expression in wild-type mice but the same effect is diminished in the hippocampi of *Lsamp* deficient mice. **(B)** *Syp* mRNA expression does not change in response to environmental enrichment. ^*^p < 0.05 (Main effect of environment in ANOVA).

The expressional analysis of the well-studied biomarker *Bdnf* (*Brain-derived neurotrophic factor*) was used as a control for the efficacy of environmental manipulation. Levels of *Bdnf* gene were upregulated in the hippocampi of mice raised in enriched environment compared to mice raised in isolation or standard housing (Figure 3A) which is well in line with data from previous studies (Novkovic et al., 2015). If the data from different housing conditions were pooled, the expression levels of both *Lsamp* transcripts correlated significantly with *Bdnf* expression levels in the hippocampus and frontal cortex. In the case of hippocampus, the correlations between *Lsamp* 1a and 1b transcripts and *Bdnf* transcript were significant in both genetic backgrounds (Supplementary Figure S1): in 129Sv mice the correlation between *Bdnf* and *Lsamp* 1a levels was 0.61 (*p* < 0.05) and between *Bdnf* and 1b levels 0.58 (*p* < 0.05). In B6 strain, the correlation between *Bdnf* and *Lsamp* 1a levels was 0.62 (*p* < 0.05) and between *Bdnf* and *Lsamp*1b levels 0.44 (*p* < 0.05).

The previously reported enrichment-induced increase of *synaptophysin* (Nithianantharajah et al., 2004) in the hippocampi of wild-type animals did not reach significance in our study (Figure 3B). It is possible that enrichment failed to induce *Syp* upregulation because of a late time-point of measurement. It has been shown previously that the exercise-induced upregulation of *Syp* starts to decline as soon as 15 days after the beginning of exercise (Ferreira et al., 2013). It is likely that the same (acute upregulation only during the first weeks) is also true in environmental enrichment which lasted 8 weeks in the current study.

## Discussion

Both genetic background and environment are involved in the formation of characteristics that are coded by specific genes. *Limbic system associated membrane protein* (*Lsamp*) gene modulates behavioral adaptation in social or anxiogenic environments. In the current study we found that environmental enrichment enhances the expression level of *Lsamp* 1b transcript specifically in the hippocampus in B6 mice; the same tendency existed across both mouse lines and both transcripts. The enrichment-induced elevation in *Lsamp* 1b expression in the hippocampal area was also significant when the data from 129Sv and B6 genotypes was pooled, indicating that the environmental effect persists regardless of genetic background. We did not detect any statistically relevant enrichment-induced differences of *Lsamp* expression in the frontal cortex, hypothalamus, thalamus, ventral striatum, or temporal lobe. It has been shown that social isolation affects the expression level of several genes (Benner et al., 2014; Siuda et al., 2014) and *Lsamp*-deficient mice tend to be insensitive to separation from the group (Innos et al., 2012); isolation stress had no effect on the expression levels of *Lsamp* transcripts in any of the brain areas investigated in the current study.

The highest expression level of the *Lsamp* gene (namely 1a promoter) in the hippocampal formation compared to other regions in the brain (Philips et al., 2015) was confirmed in the current study by using quantitative RT-PCR analysis. We also provided a detailed description of the anatomical distribution of *Lsamp* promoter activity in the hippocampus. 1a promoter is highly expressed in the pyramidal and granule cell layers. The overall expression level of *Lsamp* 1b promoter is evidently less intensive compared to 1a promoter, but the 1b-transcript positive cells are scattered all over the hippocampal formation. Although the anatomical distribution of X-Gal positive cells in the hippocampus reveals no clear neuronal staining, in the primary cell culture 1b promoter activity was detected only in the cells that had the morphology of neurons. Additionally, there is a discrepancy between alternative stainings as 1a staining is highly intensive in the granule cells of the DG whereas universal staining reveals only moderate signal in the DG (Figures 2A,C). The reliability of this picture is confirmed by the 1a and universal stainings made by 40 bp radioactive oligo *in situ* probes (Philips et al., 2015) and also by the universal staining provided by Allen brain atlas (http://www.brain-map.org/). The presence of four short transcripts initiated from 1a promoter (Lsamp-006, Lsamp-007, Lsamp-008, and Lsamp-009 according to ensemble.org database) expressed specifically in the DG could be a potential explanation for the somewhat discordant stainings of alternative transcripts in the DG of the mouse hippocampus.

In the DG, there are occasional 1b-positive cells in the granular zone, but the density of 1b promoter-positive cells is remarkably higher in the subgranular zone (SGZ) of the DG in the hippocampal formation which is known to be a specific area of enrichment-induced neurogenesis in adult rodents (Lois and Alvarez-Buylla, 1993; Brown et al., 2003; Peretto and Paredes, 2014). According to our current results there is a remarkable spatial overlap of the expressional activity of *Lsamp* 1b transcript and BrdU positive proliferating cells in the SGZ. However, *Lsamp* 1b is not expressed in all the newborn neurons and 1b transcript is occasionally active in the neurons that are surrounding and supporting new neurons. Currently it can be hypothesized that the complex regulation of the alternative promoters in *Lsamp* could be related to the maturation of neurons as newborn neurons from the SGZ eventually migrate to the GZ (Gage et al., 1998)—the area with intensive and ubiquitous *Lsamp* 1a transcript expression where only few cells are expressing 1b transcript. The vast majority of *Lsamp* 1b-positive cells in the primary hippocampal cultures showed a neuronal morphology, nevertheless, the precise phenotype of the cells in hippocampal sections remains to be defined in future studies. The study of alternative promoter activity is limited to transcript analysis at the moment as the 1a or 1b specific regions in the transcript encode for a signal peptide which is cleaved from a mature protein (Pimenta and Levitt, 2004), therefore it is impossible to separate these isoforms by using an antibody.

The eminent expressional density of *Lsamp* 1b transcript in the SGZ is in compliance with specific elevation of *Lsamp* 1b transcript in the hippocampal area of B6 mice reared in enriched environments suggesting that LSAMP is involved in the enrichment-induced neurogenesis and synaptogenesis. Furthermore, the involvement of LSAMP in synaptogenesis and synaptic transmission (Hashimoto et al., 2009; Qiu et al., 2010) in the hippocampal neurons has been shown in previous studies. The basal synaptic transmission in *Lsamp* deficient mice is not affected but CA1 long term potentiation (LTP) in *Lsamp* −/− slices has been shown to be significantly reduced suggesting that loss of LSAMP results in altered synaptic transmission and impaired plasticity in adult hippocampus (Qiu et al., 2010). As the previous evidence points that LSAMP serves as an adhesion molecule that is implicated in target recognition during synaptogenesis and also in integrity and stability of the synapses, we suggest that the enrichment-induced elevation of *Lsamp* in the hippocampal area is related to promoting synaptic connections in newborn neurons.

The baseline/control levels of *Synaptophysin* transcript do not differ in the hippocampi of *Lsamp*-deficient mice and their wild-type littermates. The elevation of BDNF specifically in the hippocampus is one of the most extensively described molecular changes (Kazlauckas et al., 2011; Kuzumaki et al., 2011; Chourbaji et al., 2012) induced by environmental enrichment. The results of the current study confirmed that effect. BDNF is one likely mediator of the long-term effects of enrichment on the phenotype doing so by promoting neuronal survival, differentiation, and synaptic plasticity (Huang and Reichardt, 2001). The reduction of enrichment-induced *Bdnf* increase in the hippocampus of *Lsamp*-deficient mice further indicates that LSAMP could serve as a positive modulator of the BDNF regulated neuronal pathways. Enrichment-induced molecular changes and synaptogenesis in the brain are not specific to the hippocampus (Rampon et al., 2000), however as for BDNF, the enrichment-induced expressional increase of *Lsamp* transcript was evident only in the hippocampus. The synaptogenesis-inducing effect of *Lsamp* could also be specific for the hippocampus as, according to our preliminary results, we have detected no effect of *Lsamp* on the rate of synaptogenesis in the primary culture of cortical neurons (data not shown) by using identical study design with Hashimoto et al. (2009) who demonstrated synaptogenesis-inducing effect of *Lsamp* in hippocampal cell culture. The expression levels on *Lsamp* transcripts correlated significantly with *Bdnf* expression levels in the hippocampus and frontal cortex, further suggesting a functional relationship between *Lsamp* and *Bdnf*.

Several expression differences were found between the mouse strains. In the hippocampus of 129Sv mice *Lsamp* 1a promoter had significantly higher expressional activity compared to B6 mice. 1a transcript has been shown to be strongly correlated with behavioral parameters associated with higher anxiety by Philips et al. (2015). Hippocampus has also been found to modulate anxiety-related behaviors by other authors (Fournier and Duman, 2013). Dorsal hippocampus was shown to be necessary for contextual fear encoding (Kheirbek et al., 2013); inactivation of ventral hippocampus has been indicated to reduce anxiety (Bannerman et al., 2004). Therefore, it can be hypothesized that the significant elevation of *Lsamp* 1a in the hippocampi of anxious 129Sv can be related to the highly anxious phenotype they show when compared to B6 mice. *Lsamp* 1b (that is more specific to sensory systems) was higher expressed in the thalamus and frontal cortex of B6 mice. This may help to explain well-known differences in physical activity, spatial memory and coordination between these mouse lines (Voikar et al., 2001; Heinla et al., 2014). It is important to note that the activation of ventral hippocampus has also been found to be anxiolytic in novel environments (Kheirbek et al., 2013). In the current study, the expression differences of *Lsamp* were not analyzed longitudinally; this discrimination could be made in the future studies. Another consideration is that newborn neurons that migrate to the DG initially exhibit increased excitability and may have distinct and yet unknown functions regarding emotional behavior (Fournier and Duman, 2013).

The key function of adult neurogenesis is to shape hippocampal connectivity according to individual needs, thereby improving adaptability over the course of life and providing evolutionary advantage (Freund et al., 2013). In our study we provide evidence indicating that LSAMP has a role in plasticity. Our data suggests that the enrichment-induced elevation of neural adhesion molecule *Lsamp* in the hippocampal area is related to promoting synaptic connections and integration of newly born cells into functional circuits. Furthermore, we provide evidence that *Lsamp* modulates enrichment-induced activation of *Bdnf* as in the absence of *Lsamp* the enrichment-induced elevation of *Bdnf* in the hippocampus is significantly reduced. We conclude that the LSAMP protein, which promotes integrity and stability of the synapses and guides target recognition of neurites in the brain, plays a crucial role in forming and rearranging connections in the hippocampus that are necessary for adapting to changes in the environment.

### Conflict of interest statement

The authors declare that the research was conducted in the absence of any commercial or financial relationships that could be construed as a potential conflict of interest.
