# Supplementary material for: Gene expression patterns and environmental enrichment-induced effects in the hippocampi of mice suggest importance of Lsamp in plasticity
Source: Front Neurosci. 2015 Jun 8;9:205. doi: 10.3389/fnins.2015.00205 (PMC4470440; doi:10.3389/fnins.2015.00205)
Supplement: Supplementary file 1 [file Image1.PDF]

Supplementary Material

Gene expression patterns and environmental enrichment-induced effects in the hippocampi of mice suggest importance of Lsamp in plasticity

Indrek Heinla1*, Este Leidmaa1;2, Karina Kongi1, Airi Pennert1, Jürgen Innos1, Kaarel Nurk1, Triin Tekko1, Katyayani Singh1, Taavi Vanaveski1, Riin Reimets1, Merle Mandel1, Aavo Lang1, Kersti Lilleväli1, Allen Kaasik1, Eero Vasar1, Mari-Anne Philips1

1Institute of Biomedicine and Translational Medicine, University of Tartu, Tartu, Estonia
2Max Planck Institute of Psychiatry, Munich, Germany
* Correspondence: Indrek Heinla, University of Tartu, Institute of Biomedicine and Translational Medicine, Ravila 19, Tartu, 50411, Estonia.

1. Supplementary scatterblots of BDNF and *Lsamp* promoter expression in hippocampus

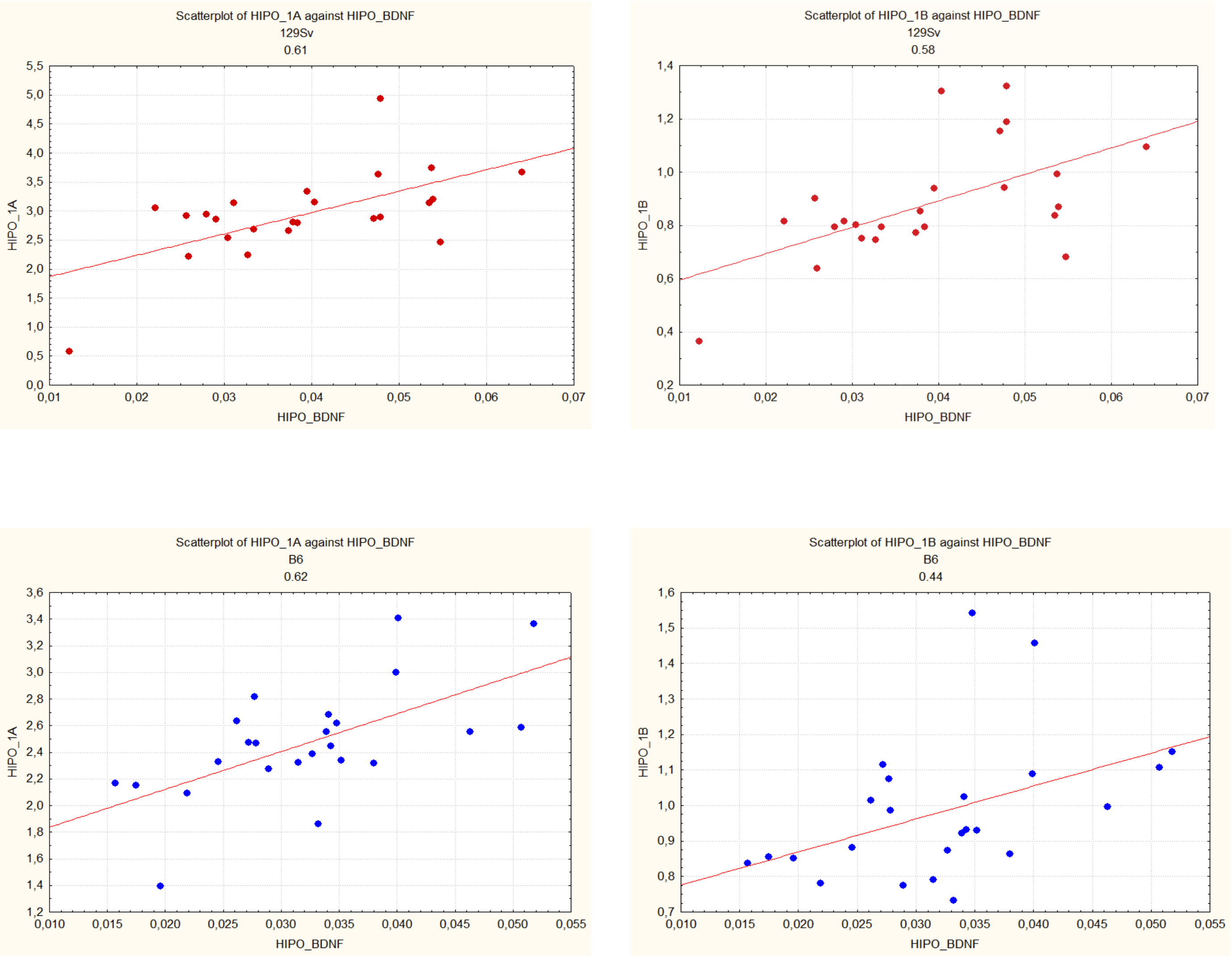

In 129Sv mice the correlation between Bdnf and Lsamp 1a levels was 0.61 ($p<0.05$) and between Bdnf and 1b levels 0.58 ($p<0.05$). In B6 strain, the correlation between Bdnf and Lsamp 1a levels was 0.62 ($p<0.05$) and between Bdnf and Lsamp1b levels 0.44 ($p<0.05$).

2. Supplementary scatterblots of BDNF and *Lsamp* promoter expression in frontal cortex

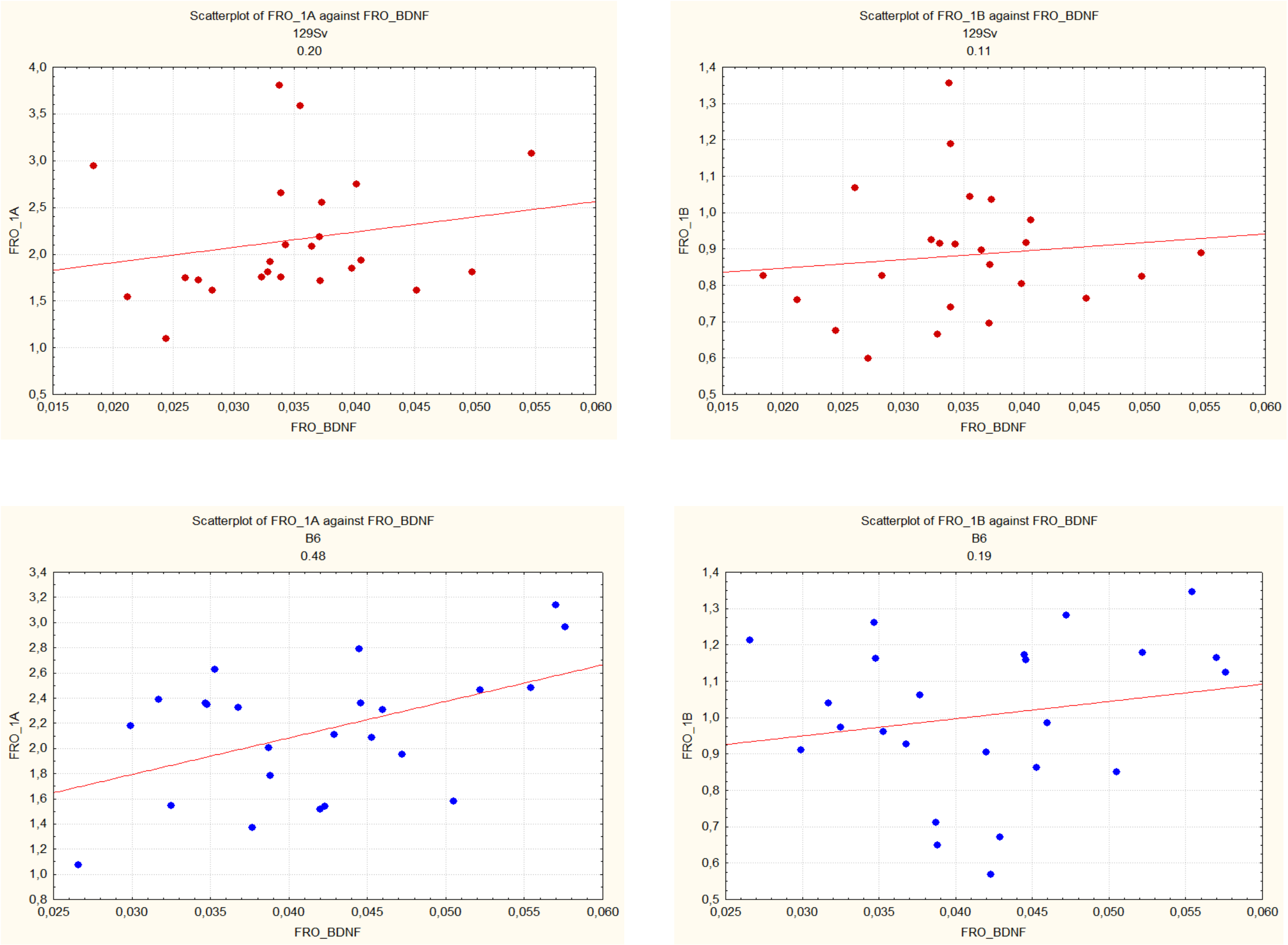

In 129Sv mice the correlation between Bdnf and Lsamp 1a levels was 0.20 and between Bdnf and 1b levels 0.11. In B6 strain, the correlation between Bdnf and Lsamp 1a levels was 0.48 ($p<0.05$) and between Bdnf and Lsamp1b levels 0.19.
